# Terahertz Spectroscopy and Imaging: A Cutting-Edge Method for Diagnosing Digestive Cancers

**DOI:** 10.3390/ma12091519

**Published:** 2019-05-09

**Authors:** Mihai Danciu, Teodora Alexa-Stratulat, Cipriana Stefanescu, Gianina Dodi, Bogdan Ionel Tamba, Cosmin Teodor Mihai, Gabriela Dumitrita Stanciu, Andrei Luca, Irene Alexandra Spiridon, Loredana Beatrice Ungureanu, Victor Ianole, Irina Ciortescu, Catalina Mihai, Gabriela Stefanescu, Ioan Chirilă, Romeo Ciobanu, Vasile Liviu Drug

**Affiliations:** 1 Pathology Department, Grigore T. Popa University of Medicine and Pharmacy of Iasi, 700051 Iasi, Romania; mihai.danciu@umfiasi.ro (M.D.); irenespiridon@yahoo.com (I.A.S.); loredana.ungureanu81@gmail.com (L.B.U.); ianole.victor@gmail.com (V.I.); 2 Medical Oncology-Radiotherapy, Grigore T. Popa University of Medicine and Pharmacy of Iasi, 700051 Iasi, Romania; 3 Department of Biophysics and Medical Physics—Nuclear Medicine, Grigore T. Popa University of Medicine and Pharmacy of Iasi, 700051 Iasi, Romania; cipriana.stefanescu@umfiasi.ro; 4 Advanced Research and Development Center for Experimental Medicine (CEMEX), Grigore T. Popa University of Medicine and Pharmacy of Iasi, 700051 Iasi, Romania; gianina.dodi@umfiasi.ro (G.D.); bogdan.tamba@umfiasi.ro (B.I.T.); cosmin-teodor.mihai@umfiasi.ro (C.T.M.); gabriela-dumitrita.s@umfiasi.ro (G.D.S.); andrei.g.luca@umfiasi.ro (A.L.); 5 Gastroenterology Department, Grigore T. Popa University of Medicine and Pharmacy of Iasi, 700051 Iasi, Romania; catalinamihai@yahoo.com (C.M.); irinaciortescu@yahoo.com (I.C.); gabriela.stefanescu@gmail.com (G.S.); vasidrug@email.com (V.L.D.); 6 Environmental Health, National Institute of Public Health, Grigore T. Popa University of Medicine and Pharmacy of Iasi, 700051 Iasi, Romania; chirilaioan@yahoo.com; 7 Electrical Engineering Faculty, Gheorghe Asachi Technical University of Iasi, 700050 Iasi, Romania; rciobanu@yahoo.com

**Keywords:** terahertz tomography, terahertz spectroscopy, cancer diagnosis, screening, digestive cancer, terahertz endoscopy

## Abstract

The Terahertz’s wavelength is located between the microwave and the infrared region of the electromagnetic spectrum. Because it is non-ionizing and non-invasive, Terahertz (THz)-based detection represents a very attractive tool for repeated assessments, patient monitoring, and follow-up. Cancer acts as the second leading cause of death in many regions, and current predictions estimate a continuous increasing trend. Of all types of tumors, digestive cancers represent an important percentage and their incidence is expected to increase more rapidly than other tumor types due to unhealthy lifestyle habits. Because it can precisely differentiate between different types of molecules, depending on water content, the information obtained through THz-based scanning could have several uses in the management of cancer patients and, more importantly, in the early detection of different solid tumors. The purpose of this manuscript is to offer a comprehensive overview of current data available on THz-based detection for digestive cancers. It summarizes the characteristics of THz waves and their interaction with tissues and subsequently presents available THz-based technologies (THz spectroscopy, THz-tomography, and THZ-endoscope) and their potential for future clinical use. The third part of the review is focused on highlighting current in vitro and in vivo research progress in the field, for identifying specific digestive cancers known as oral, esophageal, gastric, colonic, hepatic, and pancreatic tumors.

## 1. Introduction

**Figure 1:**
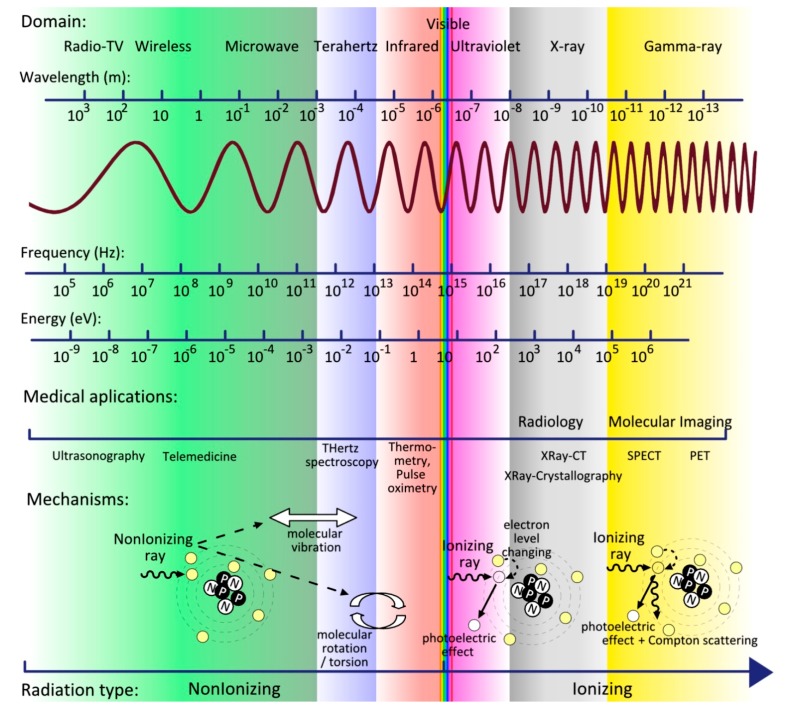
The spectrum of electromagnetic waves, characteristics, and medical applications.

The term “terahertz radiation,” which is also recognized as THz waves, T-rays, T-lux or THz light, (1 THz corresponds to 10^12^ Hz, 33.3 cm^−1^, 4.14 meV, with an wavelength of 300 μm) is located between the high frequency microwave region and long-wavelength far-infrared region of the electromagnetic spectrum as presented in Figure 1. The region is classically defined as 0.1–10 THz and was formerly acknowledged as the THz gap due to numerous difficulties related to sources and detectors [1]. The numerous molecular vibrations present in the THz frequency region (e.g., molecular rotational, torsional, crystalline phonon, intramolecular, and intermolecular) highlight the importance of crossing this gap [2].

The first notable advancement toward bridging the THz gap was made in 1975 at AT&T Bell Laboratories, when David Auston introduced stable, ultrafast femtosecond laser sources to produce and identify transmitting THz pulses [3]. Following the progress in the THz time domain spectroscopy (THz-TDS) and imaging methods, the first reported THz image dated back to 1995 [4], generated a great deal of interest, and expanded rapidly, to the point that it now facilities many areas from fundamental science [5] to tangible biomedical applications.

The dynamic character of the field is reflected in the number of recent publications. For example, a recent query on the search engine Scopus turned up barely 47093 entries for “terahertz” term, with an increasing trend from 1974 with one publication up to 4583 in 2018 and already 1079 in 2019.

## 2. THz Attractive Characteristics and Interactions with Tissues

The unique spectral features of THz radiation, namely non-ionizing, non-invasive, phase-sensitive to polar substances, spectral fingerprinting, relatively good resolution (less than 1 mm), coherent detection properties, and penetration capabilities makes THz technology particularly interesting and it has applications in spectroscopy, sensing, and imaging. The attractive characteristics of THz radiation are described below.

-*Non-invasive and non-ionizing properties* [6]: the low energy photons of THz radiation that ranges between 0.4 and 41 meV do not causes an ionization hazard, unlike X-rays.

This is an advantage in terms that it is low enough not to ionize biomolecules from the interfered tissue [7] and not to have significant thermal effect or to determine electronic transition spectra. According to Yang et al. [6], the non-invasive imaging procedure could be enforced for in vivo real-time diagnosis but also for regular screening and monitoring of patients. This hypothesis is based on several investigations on safe levels of exposure accomplished on basal keratinocytes [8], blood leukocytes [9], and human mammalian cells [10].

It is crucial to note that, regardless of being a non-ionizing radiation, THz radiation may become a biological hazard and that diagnostic applications should be made under controlled radiation power density and exposure time [11]. Furthermore, the combination of Backward Wave Oscillator (BWO) THz source as used by Borovkova et al. [11] with superlattice multipliers are a crucial emerging technology for the low energy side of the THz spectrum and should become even more relevant by replacing the BWOs with compact superlattice electronic devices (SLEDs) [12,13].

-*Phase-sensitive to polar compounds*, namely water and body fluid levels, exhibits strong absorption and better contrast than X-rays.

Studies in THz have revealed that water exhibits strong absorption at THz frequencies (α = 200 cm^−1^ at 1.0 THz) [14]. Therefore, cells and tissues with different water content display distinctive responses to THz radiation. Due to the intermolecular bonds, THz imaging can reveal hydrogen bond orientation [15]. THz radiation is diminished when it travels through media with an increased water concentration. As such, it may differentiate small fluctuations in tissue water content and in blood flow, which is a feature that makes THz attractive for monitoring and identifying different types of tumors [16] since tumors have been exposed to incorporate different water amounts than healthy tissue. In addition, THz radiation is able to promptly evaluate the living state of bacteria (live or dead) based on their altered hydration levels [6].

-*THz spectral fingerprinting*: unique spectral feature used to recognize molecules in the THz range by assessing their specific spectral signatures [6].

The energy of rotational and vibrational transitions of molecules determines specific spectra of the intermolecular interactions (such as hydrogen bonds), as well as small molecular alterations in the structure or concentration of specific protein markers in cancer. Registered through THz spectroscopy, these spectra give molecular recognition and dynamic functional evidence. The hydrogen bond distribution can also be revealed by THz imaging [16]. Additionally, the THz signal is also influenced by structural changes and disruptions determined by cancer progression [17] or by biochemical profiles of specific cancer-overexpressed proteins.

-*High spatial resolution capabilities*: THz radiation provides both time-resolved analyses of the collective vibration modes of biomolecules on the sub-picosecond to picosecond periods and spatial resolution of specific micrometers by near-field spectroscopic techniques.

Over the years, impressive efforts were committed to enhancing the THz systems resolution of following both algorithm and optics-based methods. This includes mathematically modeled transmission Point Spread Functions or different resolution and quality improvement techniques (filters in time and frequency-domains) in order to accomplish the maximum possible quality and resolution [18].

-*Coherent detection*: accurate measurements of the refractive index and absorption coefficient of samples by simultaneous calculations of the amplitude and phase of the THz radiation [19].

-*Ability to penetrate nonpolar molecules*: one of the most attractive characteristic of THz radiation that enables the detection of materials, which are opaque in visible and near-infrared domain but transparent in the THz range [6]. That high absorption coefficient promotes strong and distinctive contrast in medical imaging among materials with reduced or increased levels of water content [15].

Overall, the low-energy non-destructive interaction between THz radiation and different materials, together with the ability to be transmitted through a variety of media may give THz the potential to become an attractive and innovative imaging diagnostic method [20].

Although initially THz radiation could not be properly explored due to specific and sensitive sources and detectors absence [21,22], recent development of THz-TDS and THz imaging [23,24] has marked a milestone for identifying further potential of THz applications. Table 1 summarizes the potential of THz technologies in the pharmaceutical industry, molecular structure, molecular spectroscopy, dermatology, oral healthcare, medical imaging, and oncology. Promising results of THz detection have been obtained in the examination of gastrointestinal tract tumors. Some studies suggest that THz spectroscopy may be a potential screening technique for colorectal, hepatic, and gastric carcinoma [25,26,27].

The biomedical claims of THz technology raise several limits discussed by Yang et al. in their review [6] and briefly explained below.

The fundamental principle of THz detection is based on its property to interact differently depending on the water content of a certain environment, which suggests that water content is a key contrast agent with a stronger THz signal [6]. Even if many authors implemented different models either to simulate the dielectric function [2], or to measure the refractive index and absorption coefficient of the sample [28], the main challenge still remains to be understood of the contrast origin for a precise detection and accurate differentiation. According to Yang et al. [6], when the water absorption interferes with the measurement, sample pretreatments or microfluidic devices could be applied to minimize this issue. Additionally, the small structural differences between the reference material and the sample [6,25,26,28,29,30] also contribute to contrast the THz image. Small variations in samples are characterized by a unique and independent spectral signature, which makes THz ideal for identifying different molecules and materials.

According to Parrott et al. [2], THz spectroscopy has the capability to obtain vibrational spectra for biological systems in an aqueous phase. However, this ability shields several of the protein conformational modes due to the strong interaction of THz radiation with water. The same review considers that this difficulty will be resolved in time by understanding the weak force interactions role that occur through ligand binding in the THz absorption characteristics.

As already discussed in a previous paragraph, different concerns about the biological effects of THz radiation were elevated. Even if there are several reports on the safety levels of THz radiation that guarantee the safe use of it, different parameters should take into account power density [6,11], exposure time [11], and experimental conditions that are still a remained challenge. Additionally, the development of a standardized risk assessment sheet for experimental studies should become a priority for researchers working in the field of THz radiation.

Another important issue is represented by the relatively high cost of THz systems that blocks the routine and continuous use in real life applications. Hopefully, further development in this area will overcome this impediment and will facilitate clinical applications.

From our point of view, in our experimental studies, the most important obstacle is data analysis and interpretation, quantitative analysis, and the lack of a spectral database. Different solutions could be employed such as molecular dynamics or numerical simulations, chemometrics, or other theoretical models, in order to elucidate the interactions between samples and THz radiation and improve data interpretation, as mentioned by Yang et al. [6]. The development of a standardized spectral database will absolutely become a historic innovation in the THz field.

## 3. Integrating THz-Based Technologies with Current Diagnosis Techniques

### 3.1. THz Spectroscopy

The THz-TDS uses short pulses of THz radiation for the identifying molecules and compounds and deep structural changes, over the frequency range 0.2–4 THz in transmission, reflection, and attenuated total reflection (ATR) modes. The basic principle of the THz-TDS is based on a direct measurement using laser beam technology of both the amplitude and phase information of the THz pulse simultaneously as the function of time that is further Fourier transformed to obtain the spectra [3,19].

This method can be used in DNA studies for nucleic acid detection. It can differentiate between the four nucleobases that have different absorption coefficients in the THz range and can be used for discriminating between single-strand and double-strand DNA [35] or between hybridized and denatured DNA [36]. THz biomedical applications also include the detection of amino acids, carbohydrates, proteins, and peptides [6]. THz spectroscopy is related to THz imaging and is mainly used to define the optical properties in the frequency domain [26].

The main limitations of THz spectroscopy are that only small areas can be analyzed through spectroscopy, and it cannot accurately distinguish between two substances with similar THz spectra [80].

### 3.2. THz-Tomography

Tomographic slices are employed to recreate a certain object/tissue in 2D or 3D representation in order to assess its internal characteristics [81]. Currently, there are several tomographic techniques that use THz radiation, including, but not limited to, THz diffraction tomography, THz tomosynthesis, time-of-flight pulsed imaging, 3D THz holography, and THz computed tomography [20]. THz computed tomography (CT) uses pulsed THz radiation to provide sectional images of 3D entities similar to the conventional X-ray CT [82]. However, there are several challenges due to its high cost and technical characteristics that limit the maximum thickness of samples, and also prolong the acquisition time [20].

### 3.3. THZ-Endoscope

Currently, in vivo THz detection is limited only to the examination of superficial tissues or tumors close to the epithelial layer (i.e., breast). However, significant efforts have been made to extend THz’s applications to the diagnosis of more profound tissues and organs. In 2009, Ji et al. [83] developed a miniaturized fiber-coupled THz endoscope structure that generates and detects THz waves using an optical fiber linked with a femtosecond laser, close to the reflective surface of an organ through the excitation of the detector and generator [83]. The researchers tested the device by assessing THz reflections of the mouth and tongue and found that, at that time, the moisture of internal organs is a significant confounding issue.

Some years later, an innovative THz prototype with single-channel detection based on flexible metal-coated THz waveguides and a polarization specific exposure method was integrated into a commercial optical endoscope and demonstrated its value, which is successfully differentiated between normal and colonic cancer tissues [84].

### 3.4. THz Sensors Metamaterial Based

An interesting THz capability based on the high concentration of the electric field is represented by its potential for sensing the complex dielectric properties of small compounds of a different nature, whether it is chemical or biochemical [85].

A flexible label-free solution to investigate the dielectric properties in the THZ region was proposed by Yoshida et al. [86] by using a thin metallic mesh that senses the distinct transmittance modifications of a sample. Tao et al. [87] proposed a THz paper-based metamaterial sensor that offers distinctive resonance shifts due to the electromagnetic resonant responses at predefined frequencies for increased sensitivity and quantitative analysis of biochemical compounds. Another approach for ultrasensitive sensing of biomolecules proposes a THz sensor based on a graphene metamaterial layer that boosts the absorption of samples and tunes the sensing domain by changing the Fermi energy [88].

### 3.5. Enhancing THz Contrast

Recent studies have indicated the use of gold nanoparticles [18,89] or metamaterials with a high refraction index [89,90] in order to enhance the contrast in THz spectroscopy. Thus, this possibility arises to create a characteristic THz fingerprint by measuring the absorption coefficient and refractive index of a specific tissue [91].

## 4. Applications of THz Imaging and THz Spectroscopy in Digestive Cancers

After more than a century of research and remarkable advances in medical technology, cancer is still an unresolved dilemma. It is the second leading source of mortality in many regions, with 8.2 million deaths per year and 14 million new cases every year [92].

Currently, there are a variety of imaging methods used for diagnosis, staging, and treatment of digestive cancers such as conventional colonoscopy/endoscopy, X-ray computer tomography, magnetic resonance imaging (MRI), optical coherence tomography (OCT), and positron emission tomography (PET). However, each imaging technique has certain advantages but also disadvantages such as cost, lack of specificity, invasiveness, or radiation exposure, as presented in Table 2.

A new area in the electromagnetic radiation spectrum holds great promise for the development of medical imaging. THz can achieve an energy transfer in relation to tissue structure, providing information that can characterize its structure, with little to no harm or invasiveness [98]. Different types of electromagnetic radiation are used in several imaging methods for cancer diagnosis. Gamma radiation has revolutionized the diagnosis and the follow-up of cancer treatment (e.g., ^18^F FDG PET). In addition, fused images (PET-CT/PET-MRI) have brought the benefit of both spatial resolution and a molecular functional character of images and are considered the imaging gold-standard in many types of cancers.

A growing body of evidence indicates that THz could represent a useful instrument for identifying carcinomas at an early stage, starting from the fact that the malignant tumors have a greater content of water that strongly absorbs the THz radiation [22,24]. Additionally, THz imaging could be used for assessing if an excised tissue has tumor-infiltrated margins or not [79].

### 4.1. Oral Carcinoma

Sim et al. used THz endoscopy to assess oral cancer [99]. The authors evaluated seven samples from patients diagnosed with oral cancer and confirmed that the THz endoscope is a valid tool when it comes to distinguishing between cancerous and normal cells. The differentiation between these cells was significantly easier and more precise if THz imaging was performed in frozen tissue [99].

### 4.2. Esophageal Carcinoma

Ji et al. [28] analyzed using THz reflection imaging and spectroscopy, fragments of the gastrointestinal tract from the rat from peak to peak including: the esophagus, stomach, large intestine, small intestine harvested after perfusion with saline, and use of the refraction of THz in water as a reference. The spectroscopic analysis showed a smaller refraction index and smaller absorption coefficient in squamous epithelium from the esophagus when compared with glandular epithelium of the large intestine and stomach, which indicates a possible higher accuracy of the technique for diagnosis of esophageal compared with gastric and intestinal carcinoma [28].

### 4.3. Gastric Carcinoma

Few studies used THz spectroscopy to identify gastric carcinoma. In theory, the diagnosis may be quite difficult due to its anatomy and structure with different thicknesses of tissues. It is known that the thickness of a tissue is vital for correct THz spectroscopy characterization [89].

A study performed on six specimens of gastric carcinoma harvested during surgery and kept 4 h in NaCl 9% demonstrated that gastric carcinomas have a refraction index of about 2.5, which is similar to water. In contrast, normal tissues had a refraction index of less than 2. The authors suggested that these differences are a consequence of the increased number of blood vessels and edema in the tumor [29].

Hou et al. [100] also conducted a study on gastric adenocarcinoma tissues harvested during surgery. The tissues were histopathologically processed by embedding them in paraffin and then analyzed by THz spectroscopy, compared to normal and tumoral areas. Due to physiological changes and different tissues composition, the authors obtained two absorption peaks for carcinoma with one between 0.2–0.5 THz and the other between 1–1.5 THz. The technique was shown to be reliable, with similar results when the experiment was repeated [100]. In another study, 21 paraffin blocks of advanced gastric adenocarcinoma were analyzed by THz spectroscopy, also obtaining an increased refraction index and absorption coefficient in tumor tissues [101].

In the study by Ji et al. (2014), eight fresh gastric endoscopy specimens were analyzed from peak to peak by THz spectroscopy. The spectroscopic analysis of lesions was then correlated with their macroscopical appearance. The refraction index and the absorption coefficient were increased in the tumor. However, the authors observed that injection of saline during endoscopy might have interfered with the spectroscopy analysis. Additionally, THz spectroscopy can differentiate gastric adenocarcinoma from normal tissue but not from the “signet ring cell carcinoma” [102].

### 4.4. Colorectal Tumors

The transmission of THz-TDS and continuous wave THz imaging (CWTI) were used for the absorption coefficient and refractive indices assessment in formalin-fixed and paraffin-embedded sets of human colon tissue samples [24]. Each set contained four samples: one normal and three adenocarcinomas. They found that tumors have a greater absorption coefficient and refractive indices than normal cells. In addition, the authors concluded that several parameters, such as coefficient of absorption, refractive indices, cell densities, vascular pattern, and abnormal microenvironment (O_2_ level, pH value, glucose level, and lipid concentration) have an impact on the results [24].

Wahaia et al. (2015) analyzed 30 paraffin-embedded blocks of human colon tissue samples (normal and tumor) (11 pT3 adenocarcinoma and 10 pT4 adenocarcinoma) [30,103] through multipoint transmission THz-TDS. The aim of the previously mentioned work was to evaluate if cell alterations, abnormal density alternations, abnormal vascular patterns, or cell density can alter the contrast. The authors found that pT4 adenocarcinoma vs. pT3 adenocarcinoma had a greater absorption coefficient and refractive indices [103]. The THz absorption and reflection may also be assessed in the absence of water [30].

Reid et al. (2011) conducted a study on normal tumor and dysplastic colon samples from 30 patients using a conventional THz-TDS system. The study showed a sensitivity of 82% and a specificity of 77% in distinguishing the normal from the tumor tissue and a sensitivity of 89% and a specificity of 71% in differentiating the normal from the dysplastic one [27]. Another study, using diverse intelligent analysis methods (neural networks, decision trees, and support vector machines) re-evaluated the data provided by Reid et al. This method increased the sensitivity to 90–100% and the specificity to 86–90%, which improves the overall precision of the diagnosis [25].

Furthermore, continuous-wave polarization imaging THz was used on 14 fresh 3–5 mm thick human colonic samples from eight patients enveloped in a wet compress with a 7.4 pH. The results showed good correspondence between THz imaging and histology [104].

### 4.5. Hepatocarcinoma

Several studies have been published regarding THz imaging and liver, with methods including ex vivo analysis of fresh, formalin fixed, and paraffin embedded (FFPE) liver tissue.

A study of freshly excised human liver tissue with hepatocellular carcinoma placed the absorption coefficient at about 9 to 12 mm^−1^ for normal liver tissue, while cancer tissue had a coefficient larger than 12 mm^−1^. The authors compared the THz near-field images with the corresponding pathological examination slides and concluded that THz allows for accurate identification of normal and abnormal tissue [105].

Since there is no standardized method for measuring the amplitude and refractive spectra under THz radiation, the evaluation of paraffin embedded human tissue samples can be enhanced by performing the acquisition of data in a mixture of paraffin and water. This method reduces the background noise and increases the speed of image acquisition while enhancing the differences between hepatocellular carcinoma cells and normal cells [106].

Experimental models of hepatocellular carcinoma, particularly mouse and rabbit models, have been developed to reproduce different human clinical conditions. In the beginning, mouse models were created to establish a baseline of healthy hepatocytes in fresh liver tissue by noting the changes in the state of polarization of the THz wave [107].

A study on a rabbit VX2 liver tissue tumor model was developed to evaluate this new technology. It showed different amplitudes for tumor cells, healthy cells, and paraffin areas, which creates a sharp delineation between healthy and cancerous tissue. These images were well correlated with the pathological hematoxylin and eosin images. The study also identified regions of low correlation, corresponding to areas of tumor necrosis [108].

The transmission of THz-TDS is qualified for extracting amplitude and phase parameters of the pulsed signal and can also map the image of the tissue pixel by pixel. However, it is rather slow with regard to the acquisition time and requires rather large tissue samples. In contrast, THz digital holography can acquire multi-plane information of a target in the THz range in a shorter amount of time, with improved structural details [109,110]. It can contribute to the early diagnosis of hepatocellular carcinoma by identifying the variations in water content and the presence of fibrosis in the liver [111].

### 4.6. Pancreatic Cancer

Due to the anatomical location of the pancreas, studies involving pancreatic tumors are scarce. A study performed by Brun et al. (2010) used 10-µm-thick tissue samples that were processed in a water solution. Images were obtained through both digital slides processing as well as THz imaging techniques. The results demonstrated that the THz method can accurately differentiate between normal and cancer cells, can assess the degree of invasiveness, and can even identify two distinct tissue subtypes in the tumor area [79].

## 5. Team’s Preliminary Results in THz Field

Our contribution to the THz domain is, for the moment, divided in two sections.

(A) The first step involves the development and evaluation of the tailored nanoparticle as THz molecular imaging contrast agents for early-stage diagnostic of gastric neoplasia. In this sense, Fe_3_O_4_ and Gd_2_O_3_ nanoparticles were functionalized by citric acid and carboxymethylcellulose and were tested in vitro and in vivo through specific tests in order to evaluate their capability as viable contrast agents. The most adequate formulations for nano-assemblies as contrast agents were established through validation of contrast efficiency, along with successful stealth properties and functional tests. The cell viability tests on standardized human gastric cell line AGS of different nanoparticle concentrations revealed a dose-dependent effect in 24-h determinations [112]. The in vivo studies on mice revealed adequate biocompatibility of the administered nano-assemblies when compared to the control batch (data not published). In the next step, the magnetite nanoparticles stabilized in citric acid and carboxymethylcellulose were evaluated as THz imaging contrast agents in gastric cancer cell lines. The preliminary results of THz images obtained at 1.1 THz showed consistent differences between normal and cancerous tissue. The influence of the optical effect of the nanoparticles on the frequency domain of 1.1 THz was clearly observed (data not published).

(B) The second step implicates the elaboration of several solutions for THz imaging limitations. It is well known that THz imaging has a poor signal-to noise ratio, slow processing, and low-brightness. Therefore, the development of advanced image processing software may lead to an important breakthrough in early detection of a gastric cancer application [113]. The authors concluded that the development of the image processing software for THz imaging must ensure the following parameters: versatility vs. frequency, adaptability to transmission or reflexion measurements, provision of accuracy and selectivity of images with control on image quality, and reduction of the irregularities of received data, the possibility to increase the visibility of fine details in an image by adjusting pixel intensities, and expansion of smaller regions on predefined criteria for cancer detection, along with selective 2D and 3D visualization options of the rendered object [113].

Further work will follow to overcome the existing limitations in THz imaging and spectroscopy and to find new innovative solutions in clinical applications.

## 6. Conclusions

THz-based technology is a promising and innovative new tool for medical diagnosis. Although remarkable advancement has been attained in THz detection and tissue imaging, there are still several issues that hinder its large-scale use such as high costs, lack of sufficient discriminative precision, data analysis, and interpretation. However, taking into account the fact that THz procedures and devices are not yet fully developed and are continuously under research and expansion and that THz is being integrated into smaller and smaller medical devices, it is safe to assume that these obstacles will be controlled in several years. Due to its unmatched sensing as well as non-invasive and non-destructive features, this type of radiation can potentially replace other forms of radiation used for the diagnosis and monitoring of different types of conditions.

## Figures and Tables

**Table 1:** Overview of the biomedical applications of THz technology.

Class	Technique	Detected Compounds	References
Nucleic acids	THz-TDS	DNA, sequences of oligonucleotides, RNA	[31,32,33,34]
	THz photo-mixing	DNA	[35]
	THz-based metallic mesh	DNA: single-stranded and double-stranded	[36]
	Time-resolved THz	Hybridized/denaturated DNA films	[37]
Amino acids and peptides	THz-TDS and transmittance spectroscopy	20 naturally occurring lyophilized amino acids, 1-serine and 1-cysteine, _L_-cysteine and _L_-histidine, polyglycine and poly-_L_-alanine, _L_-glutamic acid, Histidine analogs of oxytocin and vasopressin, Alanine-rich peptides, _L_-Threonine and glycine	[38,39,40,41,42,43,44,45]
Proteins	THz-TDS	Wild-Type and D96N mutant Bacteriorhodopsin, Photoactive protein systems: rhodopsin and bacteriorhodopsin, Hen egg white lysozyme, Immunoglobin (IgG) protein, Protein hydration and protein–ligand binding, Protein–ligand binding among hen egg white lysozyme and triacetylglucosamine	[46,47,48,49,50,51]
Tissues (dermatology)	THz-TDS	Human scleral tissues, Rabbit corneal tissue ex vivo, Skin tissues vs. human basal cell carcinoma, Bone, Brain tissues from Alzheimer disease patients	[52,53,54,55,56]
	THz empirical mode decomposition	Fresh porcine muscle and skin tissues	[57]
	THz pulsed imaging	Rat tissues, Basal cell carcinoma and melanoma, Porcine skin burns, Human skin in vivo (stratum corneum thickness and hydration)	[58,59,60,61]
Dental health	THz pulse and reflection imaging	Teeth tissues, 32 human permanent teeth surfaces (detection of dental caries ex vivo), Enamel demineralization in vitro.	[62,63,64]
	THz-TDS	Enamel-dentine boundary	[65]
Pharmaceuticals	THz pulse imaging	Tablet and coating integrity and performance	[66]
	THz Spectroscopy and THz-TDS	Direct measurement of crystallization and molecular mobility of amorphous pharmaceuticals. Pharmaceutical materials and tablets. Aspirin and Aspirin Precursors. Pharmaceutical Polymorphism and Crystallinity. L-, D-, and DL-Tartaric Acid.	[67,68,69,70,71,72]
Oncology	THz pulse and reflection imaging	Skin cancer: ex vivo and in vivo basal cell carcinoma, nonmelanoma. Breast cancer: in situ non-calcified form, triple negative infiltrating ductal carcinoma, micro-metastatic lymph nodes. Lung neoplasm: squamous cell carcinoma. Uterine cervical neoplasm.	[73,74,75,76,77,78,79]
	A specific discussion on digestive cancers can be found in following sections.		

**Table 2:** Comparative performance parameters of imaging diagnostic methods.

Technique	Spatial Resolution, mm	Damage to Healthy Tissue	Limits
Conventional colonoscopy/endoscopy	Lack of spatial resolution [93]	Invasive	Operator dependent and can have high false-negative rates
X-ray computer tomography	0.5–2 [94]	Minimal radiation exposure	Low sensitivity for digestive cancers and can not be repeated very often-
MRI	0.1–2 (depended on Tesla range) [95]	Non-invasive, uses contrast agents	High cost, time consuming investigation
OCT	0.01 [96]	Non-invasive	High cost, subjectivity in image interpretation, research use only
PET	0.54–6 (dependent on the isotope) [97]	Minimized risk tissue damage due to radiation	High-cost, the lack of anatomic correlation, restricted availability
THz technology	0.1–0.25 [21]	Non-invasive and non-ionizing under controlled radiation power density and exposure time	As discussed in a previous section
